# Plant community dynamics of lomas fog oasis of Central Peru after the extreme precipitation caused by the 1997-98 El Niño event

**DOI:** 10.1371/journal.pone.0190572

**Published:** 2018-01-02

**Authors:** Carolina Tovar, Edgar Sánchez Infantas, Vanessa Teixeira Roth

**Affiliations:** Departamento de Biología, Facultad de Ciencias, Universidad Nacional Agraria La Molina, Av. La Molina s/n, Lima, Peru; GIScience Department, University of Jena, GERMANY

## Abstract

Despite El Niño events being one of the main forces shaping the coastal desert vegetation in South America, the impacts of the high precipitation typical of this rare but recurrent climatic event remain understudied. Here we monitored the plant community of a coastal *lomas*, a seasonal desert ecosystem, during 1998 and 2001 to analyse its changes during the 1997–98 El Niño and the following La Niña events. We measured species abundance and vegetation cover in 31 plots, and recorded climate variables in Lomas de Lachay, Peru. We found a significant positive correlation between precipitation and vegetation cover, density, alpha diversity (species diversity at the plot level), total richness and abundance of several key species but no correlation with gamma diversity (species diversity at the whole loma level). During the El Niño event, the seasonality, typical of the lomas ecosystem, disappeared, as evidenced by both the similarity of species composition and mean vegetation cover values between most sampling campaigns of 1998 and 1999. Moreover, total richness was lower during the El Niño event than during the humid season of 2000 and 2001 resulting from the dominance of only a few species, such as *Nicotiana paniculata* and *Loasa urens*. Temporal-spatial changes in the abundance of the dominant species caused the differences between alpha and gamma diversity, especially during 1999. Within that year, mean alpha diversity showed similar values whilst gamma diversity values were different. The reestablishment of the seasonality of most plant community characteristics and a clear difference between species composition of the humid and the dry season occurred two years after the El Niño event, suggesting a resilient community. This study provides one of the few quantifications of the Peruvian lomas’ response to the 1997–98 El Niño event and the following La Niña, one of the most extreme climatic events in the last century.

## Introduction

Infrequent pulses of rain are important drivers of biological communities in arid ecosystems [1]. Therefore, extreme changes in precipitation patterns have a strong influence in these arid regions [2]. One of the main sources of climate variability are extreme events, such as the El Niño Southern Oscillation (ENSO), a recurrent but irregular climatic phenomenon that alternates a warming phase known as El Niño and a cooling phase known as La Niña [3–5]. This phenomenon has a worldwide impact on the climate [5,6] and while some parts of the world experience severe droughts other regions experience a dramatic increase of precipitation [3]. During El Niño events, positive rainfall anomalies occur in typically arid and semi-arid regions such as the west Coast of South America, southern United States and east Africa [2,7,8]. However, recent studies suggest the existence of various El Niño “types”, for example, the Eastern Pacific coast suffered stronger impacts from the 1997–98 ENSO than those expected from the 2015–16 ENSO despite of having similar tropical sea surface temperature anomalies [9,10]. The origin of ENSO dates back to at least 5000 years [11] and its frequency has increased in the last 3000 years [12]. The long-term occurrence of El Niño events suggests that arid and semi-arid vegetation communities may have been strongly shaped by this climatic event [2,13].

In the Peruvian and Chilean coastal deserts [14–16] and the northern Peruvian dry forest [17,18] El Niño events result in a substantial increase in vegetation within these ecosystems. In addition to the increase of primary productivity, an increased number of species has been recorded during this event [14,16,19,20] as well as a higher production of seeds [21,22]. Most of these studies focused only on the El Niño phase and very few on the subsequent La Niña phase but some long-term studies exist, mostly restricted to Chile [2,16,23]. Few studies have included surveys from the 2011 La Niña phase, accounting for the vegetation dynamics during this phase [24,25]. Therefore, there is a need to quantify long-term vegetation responses to ENSO, particularly in the Peruvian Coast, to better understand ENSO effects on the organization of the plant communities, including changes in composition, diversity and primary productivity. Given that the frequency of extreme El Niño events is likely to increase under climate change scenarios [26] a better insight into the ecological consequences in these fragile arid ecosystems will help to develop conservation and management strategies for them.

**Fig 1:**
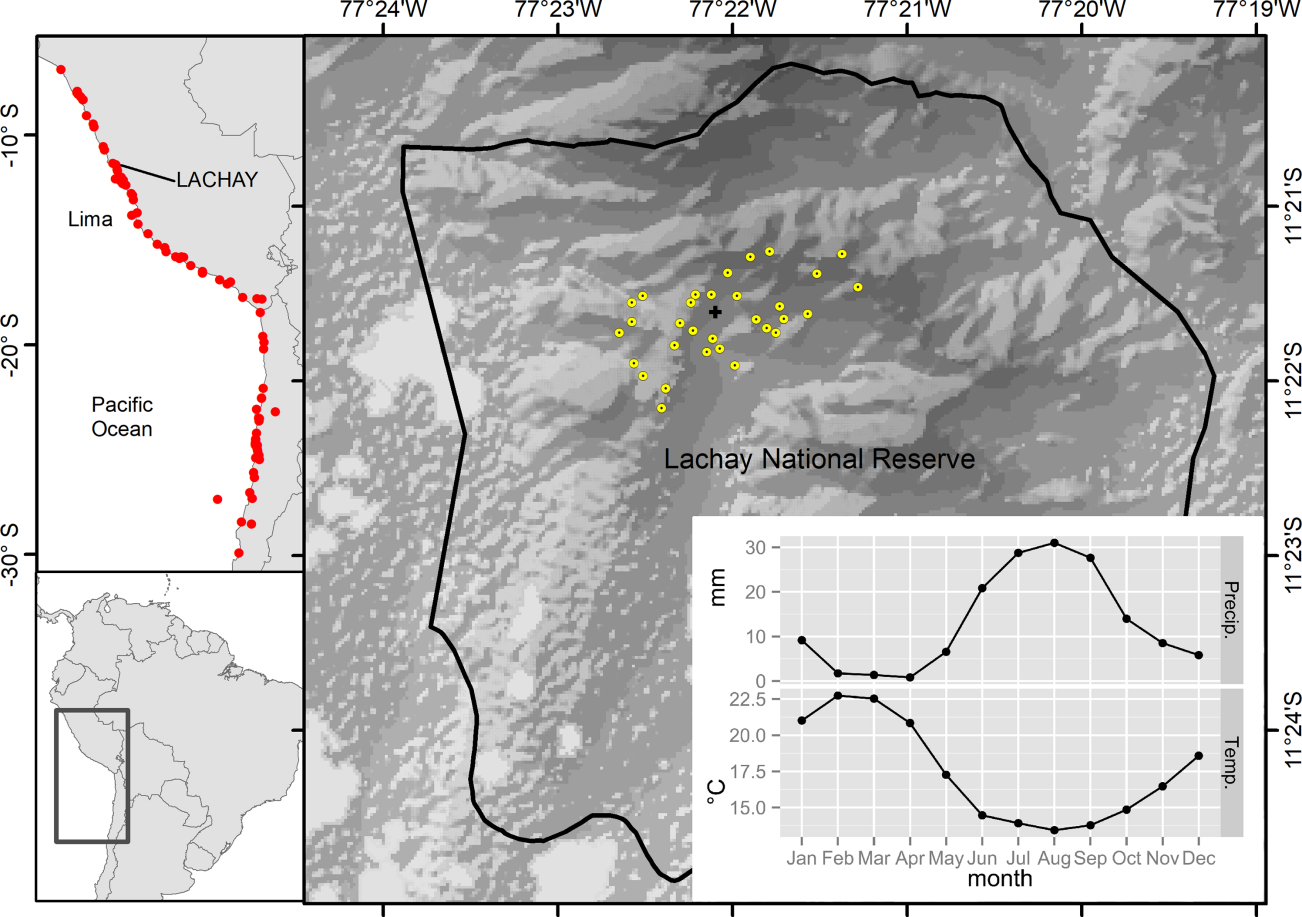
Location of the South American lomas vegetation (red dots), the study area with the location of the sampling points (yellow points) and the location of the SENAMHI meteorological station (black cross). Precipitation and temperature climatologies for Lomas de Lachay are the average values for the period 2000–2010 (SENAMHI data). The digital elevation model was retrieved from Shuttle Radar Topography Mission (SRTM) 1 Arc-Second Global (https://lta.cr.usgs.gov/SRTM1Arc).

Within the desert vegetation of the South American coast, the lomas, also known as fog oasis, are isolated vegetation communities along the Chilean and Peruvian coast (Fig 1) occurring on small mountains or steep coastal slopes (< 1000 m a.s.l.) separated by flat hyper arid desert [14]. A typical loma mostly has annual herbs, but also perennial herbs, and a few woody plant [27,28]. Small trees such as *Caesalpinia spinosa* are restricted to a few lomas [28,29]. Isolation has promoted high levels of endemism, thus, there is little overlap between the species composition of the northern Peruvian, southern Peruvian and the Chilean lomas plant communities [30]. The lomas are classified as a fragile ecosystem, mainly because its extent has reduced considerably due to human activities such as overgrazing, logging and urban encroachment [29,31,32].

The lomas is an ecosystem with two distinct seasons, a dry season and a humid season (mostly between May and October) where elevational fog condenses to provide moisture for vegetation [28,33]. However, during El Niño events, high precipitation occurs throughout the year, causing constant vegetation presence, especially of ephemeral herbs [14]. Most studies that recorded El Niño event effects on lomas plant communities have focused on quantifying species richness [14,16,19,34], primary productivity [35] or abundance [36,37] separately. Recent studies have considered a more integrative assessment of lomas plant communities [24,38]. However, there are fewer studies that evaluate multiple plant community characteristics together during the ENSO.

The aim of this study is to quantify the dynamics of the plant community of a lomas ecosystem during and after the 1997–98 El Niño event (Feb 1998-Dec 2001). This period covers part of the El Niño event (Feb-May 1998) and the La Niña event (Jul 1999–Feb 2001) [39]. We monitored species composition, vegetation cover, density, alpha and gamma diversity and total richness in the Lomas de Lachay National Reserve, Lima, Peru. We sought to answer three questions: 1) Is there any correlation between the lomas plant community characteristics and climate? 2) What are the changes in the plant community during the El Niño and La Niña events? 3) How long does it take to recover the seasonality of the plant community after an El Niño event? Given the recurrence of extreme El Niño events, we expected the plant community to be highly adapted to this event.

## Materials and methods

### Study area

The Lomas de Lachay National Reserve is located 105 km north of Lima city, Peru (Fig 1). The total area of the Reserve is 5 070 ha and the study area spans 430 ha located in the core area of the National Reserve. The Lomas de Lachay’s ecosystem experiences a rapid increase in vegetation biomass, mainly of herbaceous species between July and September (humid season). This vegetation, with the exception of sparse shrubs and trees, then recedes throughout the drier months of the year [40]. The number of species recorded in the Reserve is 146 and the dominant families are Asteraceae and Solanaceae [34]. There are a few arboreal species such as *Schinus molle*, *Caesalpinia spinosa* and *Capparis prisca*, but the herbaceous species represent 79% of the species composition while the shrub species represent 14% of the composition [34]. The mean monthly temperature ranges between 13.5°C (humid season) and 22.5°C (dry season). The total monthly precipitation ranges from 0 (dry season) to 30 mm (humid season) (Fig 1) and the mean total annual precipitation for the period 2000–2010 was 153 mm.

### Data collection

#### Sampling design

We carried out a preferential stratified sampling design. First, the study area was stratified in three subareas according to relief characteristics and tourist use zones (high: most visited area, medium: moderately visited area, low: restricted area from tourists) [41]. Second, within each subarea, the plots were located outside the visitor paths to avoid any direct human impact. We monitored 31 plots of 1m^2^ (12 in low tourist use zone, 8 in medium use zone and 11 in high use zone) distributed between 280 and 690 m a.s.l. representing areas at the hills top, medium and low elevations (Fig 1). Vegetation measurements were collected four times per year between 1998 and 2001 to record the changes in the annual seasonality with one sampling campaign taking place in each of the following periods: January-March (driest season), April-June (dry season), July-September (humid season), October-December (dry season). In 2001, we were only able to conduct three sampling campaigns due to financial and personnel constraints (Table 1). Surveys for each sampling campaign lasted between three to four days

**Table 1:** Plant community characteristics and climate data recorded between 1998 and 2001. ENSO phases are described in methods. Ranges of values are shown in brackets.

Date	Global ENSO phase (ONI)[39]	Peruvian coastal warm/cold conditions (ITCP)[42]	Mean density (ind.m^-2^)	Mean cover (cm^2^.m^-2^)	Mean α diversity	ɤ diversity	Total richness	Monthly pp (mm)
Feb-98	El Niño	Warm	75.5 (1–847)	18912 (1646–111440)	1.05 (0–2.23)	2.63	29	48
May-98	El Niño	Warm	16.9 (1–62)	7952 (1500–11000)	0.88 (0–1.85)	2.25	13	16.5
Aug-98	La Niña	Normal	37.3 (1–141)	16185 (2691–104189)	1.2 (0–2.14)	3.11	24	46.4
Dec-98	La Niña	Normal	14.1 (1–103)	9268 (1575–51977)	0.57 (0–1.50)	2.59	15	2
Feb-99	La Niña	Normal	4.4 (1–20)	5944 (122–17671)	0.64 (0–1.92)	3.11	14	4.4
May-99	La Niña	Normal	10.4 (1–57)	7176 (227–44614)	0.65 (0–1.58)	2.41	13	18.9
Aug-99	La Niña	Normal	8.8 (0–66)	4838 (0–13110)	0.84 (0–2.32)	3.1	23	18.7
Nov-99	La Niña	Normal	2.3 (0–14)	2722 (0–20000)	0.4 (0–1.66)	3.32	15	8.2
Feb-00	La Niña	Normal	2.2 (0–17)	2452 (0–8081)	0.34 (0–1.50)	2.64	11	0
May-00	La Niña	Normal	0.6 (0–2)	892 (0–5675)	0.06 (0–1)	1.98	6	6.1
Aug-00	La Niña	Normal	347.7 (4–1655)	14721 (6494–25652)	1.58 (0–2.91	3.28	41	40.2
Nov-00	La Niña	Normal	1.3 (0–6)	1489 (0–7854)	0.19 (0–1.58)	3	11	1.4
Feb-01	La Niña	Normal	0.5 (0–4)	983 (0–5675)	0.09 (0–1)	1.24	3	0.4
Sep-01	Normal	Cold	347.6 (5–1506)	11728 (1806–22762)	1.4 (0.21–2.57)	3.23	37	29.2
Dec-01	Normal	Cold	1.6 (0–13)	1304 (0–8908)	0.13 (0–1.50)	2.59	9	1.2

There were 15 people involved in the vegetation surveys over the four years. Botanical identifications were done by well-known senior botanists from Peru (see acknowledgments) which avoided potential identification bias.

#### Vegetation measurements

We counted the number of individuals per species in each plot and measured the minimum and maximum diameter of the perpendicular projection of the vegetation cover per species to calculate the area of vegetation cover (cm^2^.m^-2^) [43,44]. When individuals of the same species overlapped, we measured the perpendicular projection of the group [45]. The vegetation cover measurement was overestimated for February 1998 and August 1998 because instead of using the perpendicular projection of all individuals when they overlapped the projection was done for each individual. The values of vegetation cover for these two sampling campaigns were removed from statistical analysis where absolute values were needed but were used when the input data was relative.

In order to assess the ecological importance of the species in the lomas we calculated an importance value index (IVI_i_) per sampling campaign. This index can use one, or combinations of two or three, of the following parameters: relative frequency, relative abundance and relative vegetation cover depending on the objectives of the study [46,47]. Historically the most noticeable response of the vegetation of the South American coast to El Niño events has been the extensive blooming of the desert [48], therefore we chose to combine the relative vegetation cover and relative abundance because the first one can be interpreted as a proxy of biomass and the latter of regeneration. These two variables were weighted for each species as shown in Eq 1 per sampling campaign:

$$IVI_{i}=\frac{n_{i}/N_{t}+c_{i}/C_{t}}{2}$$ (1)

Where the first component of the right hand of the equation represents the relative abundance (n = number of individuals of the species *i*, Nt = total number of individuals of all species) and the second component represents the relative cover of each species (c = vegetation cover of the species *i*, Ct = total vegetation cover of all species). To facilitate interpretation, we divided by 2 so that the index ranges from 0 to 1. We chose to use relative values to avoid the bias created by the difference in absolute values of the two original variables. Also, as these are relative values we included the vegetation cover records of February and August 1998.

We also calculated the following plant community characteristics for each plot in each sampling campaign: 1) Density (ind.m^-2^) was estimated as the sum of all individuals of all species per plot [45], 2) Vegetation cover (cm^2^.m^-2^) was calculated by summing the vegetation cover of all species per plot. 3) Alpha diversity was estimated by using the Shannon diversity index [49] per plot using the species abundances in Eq 2. This index considers both the number of species and their relative abundances. The index equals zero when the plot contains a single species, increases with the number of species and would be maximum when all species have equal number of individuals.

$$H=-\sum_{i=1,2,\ldots ,s}p_{i}\;{log}_{2}\;p_{i}$$ (2)

where *s* = number of species, and *p*_*i*_ = abundance of a species in a plot divided by the total abundance in the plot.

Finally, we also calculated the following plant community characteristics for our entire study area: 1) Total richness was estimated as the total number of species in our study area per sampling campaign. 2) Gamma diversity was estimated by using the Shannon diversity index [49] using Eq 2 where the abundance data was that of all plots together for each sampling campaign.

#### Climate data

We obtained mean monthly temperature (°C) and total monthly precipitation (mm) between 1998 and 2010 from the meteorological station located in Lomas de Lachay (11,3601°S, 77.3683°W, Fig 1) that belongs to the Peruvian hydro meteorological service (SENAMHI). Data between 1998 and 2001 was used to test any potential correlation between the climate variables and the plant community characteristics. We averaged monthly precipitation and temperature values between 2000 and 2010 to obtain an annual time series for non-extreme El Niño years. According to NOAA, which uses the Oceanic Niño Index (ONI), the El Niño event occurred between May 1997 and May 1998 while the La Niña event occurred between July 1998 and February 2001 [39]. However, at a regional level, using the Peruvian coastal thermal index (ITCP by its Spanish name), warmer than normal conditions in the Peruvian coast occurred between March 1997 and June 1998 while colder than normal conditions were only recorded between April and June 1999, September 2001 and January 2002 [42]. We have matched these events to our fieldwork dates in Table 1.

### Statistical analysis

#### Correlation between plant community and climate variables

We calculated the Spearman correlation between plant community characteristics per sampling campaign and the climate data (temperature and precipitation) of the same month in which the campaign took place. We used the following plant community characteristics: total richness, gamma diversity and the averaged values of density, vegetation cover and alpha diversity of all plots per sampling campaign. A 95% confidence interval was used for this analysis and p-values were adjusted for multiple comparisons using the Bonferroni correction, which corrects the p-values based on the number of performed tests. We also tested for a significant correlation between the density of the 20 most abundant species across all sampling campaigns and the climate variables using the Spearman correlation and the Bonferroni correction. Analyses were performed using R software (https://www.r-project.org/).

#### Analysis of temporal plant community dynamics

First, we performed a detrended correspondence analysis (DCA), an ordination technique, to explore the major gradients in plant community composition across the different sampling campaigns. We preferred a DCA instead of a principal component analysis because the latter treats double zeros, typical of ecological data, as similarities [50]. For this analysis, we used a matrix of IVI values (see vegetation measurements) of 60 species x 15 sampling campaigns. The DCA was performed using the R package Vegan (version 2.3–0, https://cran.r-project.org/web/packages/vegan/index.html). Rare species were down-weighted. A DCA biplot was used to show the variation in floristic composition across sampling campaigns.

Second, we tested the difference between the 15 sampling campaigns for density, cover and alpha diversity using paired t-tests. We used the values calculated for each plot in each sampling campaign for this analysis. Paired t-tests are designed specifically for repeated measurements. Given the high number of multiple comparisons (105 tests), we used the Bonferroni correction. By comparing the sampling campaigns within each year, we can test the effect of changes in seasonality. Additionally, we also compared the values for the same month throughout the different years. In the case of vegetation cover we excluded the sampling campaigns of February and August 1998 due to the overestimation of the cover (see vegetation measurements section). We also checked whether there were differences in density, cover and alpha diversity between tourist use zones using the Kruskal–Wallis test (S1 Appendix).

#### Analysis of changes in vegetation seasonality

Vegetation seasonality has been mainly measured using time series analysis of satellite products to estimate the variation in greenness over a year [51,52], for example using the amplitude of the oscillations between the lowest and maximum points of greenness [53]. In the absence of adequate satellite products for our study area during the studied period (<30% clouds coverage, at least 4 images per year), we sought to represent the vegetation seasonality with the help of our field data. Therefore, we selected the sampling campaigns of the typically humid months (between July and September) and the typically driest months (between January and March) to develop our index (SI) for each plant community characteristic. This way, we captured seasonality patterns. SI was calculated as shown in Eq 3.

$$SI=\frac{\left(Value\;in\;August-Value\;in\;February\right)}{Value\;in\;August}\times 100$$ (3)

SI, basically compares the annual range between values of the most humid month (August) and the driest one (February). Larger positive values of SI would indicate a bigger difference between typically humid and dry months for a given year while values closer to zero indicate the opposite.

Negative values indicate a complete alteration of the seasonality, a scenario where the normally driest month has the highest values of density, cover, etc. We plotted the SI through time and looked for the point in time when it stabilizes after the El Niño event to evaluate how long it took for the pattern of vegetation seasonality to be re-established.

## Results

### Floristic composition

**Fig 2:**
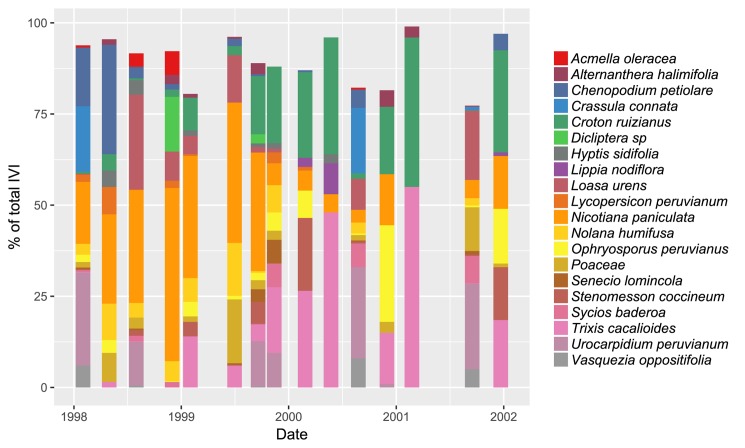
Floristic composition for each of the 15 sampling campaigns. Only the top 20 species according to their importance value index (IVI) are shown (see methods).

We recorded 60 species that belong to 29 families during the 4 years of monitoring (S1 Table). The Euphorbiaceae *Croton ruizianus* was present during all sampling campaigns, while the Solanaceae *Nicotiana paniculata* and the Asteraceae *Trixis cacalioides* were registered in 14 out of the 15 sampling campaigns (Fig 2, raw data in S2 Table). The species with the highest values of total vegetation cover during the 4 years were *Nicotiana paniculata*, *Urocarpidium peruvianum*, *Loasa urens*, *Croton ruizianus* and *Nolana humifusa*. The highest values of total density for all sampling campaigns were registered for *Urocarpidium peruvianum*, *Crassula connata*, *Loasa urens*, *Vasquezia oppositifolia*, *Nicotiana paniculata* and *Chenopodium petiolare*. Total richness per sampling campaign varied between 3 and 41 species (Table 1 and S1 Fig).

### Correlation between plant community and climate variables

The highest recorded value of precipitation was 100 mm.month^-1^ during January 1998 (S1 Fig), towards the end of the El Niño event, and it was ten times the precipitation normally observed for that month in a typical year and more than three times the precipitation for the most humid month of a typical year (Fig 1). We found significant positive correlations between total monthly precipitation and the average values of alpha diversity, vegetation cover, density and total richness of all plots per sampling campaign but not with gamma diversity (S3 Table). In the case of species density, precipitation showed a significant positive correlation with a few species such as *Senecio lomincola*, Poaceae sp1 and *Vasquezia oppositifolia* among others (See S4 Table).

Contrary to what we observed for precipitation, mean monthly temperature values during the 1997–98 El Niño event were the same as in a typical year (Fig 1 and S1 Fig) and did not have any significant correlation with any of the plant community characteristics or the species (S3 and S4 Tables).

### Plant community dynamics during and after the 1997–98 El Niño event

**Fig 3:**
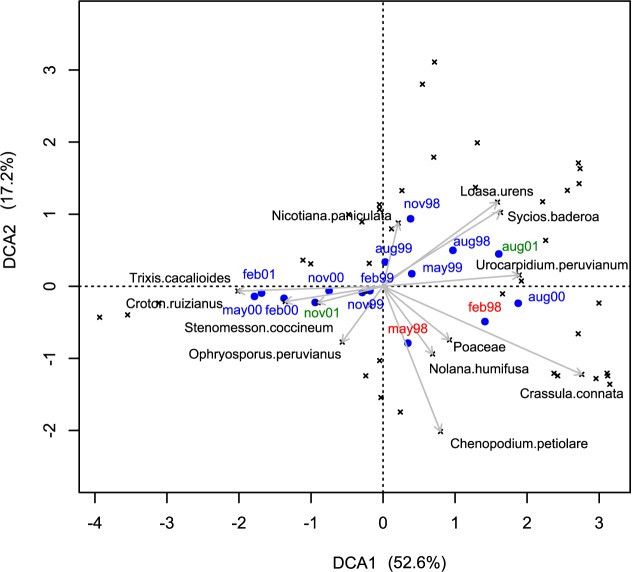
Changes in species composition. Biplot of the detrended correspondence analysis (DCA) showing species in cross and the different field sampling campaigns in dots (red = the El Niño event, blue = the La Niña event, green = normal conditions, according to NOAA [39]). For clarity, only the most important species are shown, based on the importance value index (IVI).

The change in species composition across sampling campaigns is observed in the DCA biplot (Fig 3). Together the first and the second axis represented 69% of the total variance. The first axis represents a gradient of humidity where *Crassula connata* and *Urocarpidium peruvianum*, in the right side of the biplot, were among the dominant species during the most humid fieldwork campaigns (February 1998, August 2000 and August 2001). These includes El Niño event, La Niña and a normal year respectively (Table 1). In the left side of the biplot *Trixis cacalioides* and *Croton ruizianus* were among the dominant species during the dry sampling campaigns (February, May and November 2000, February and November 2001) that represent La Niña and normal conditions. The second axis represents an El Niño-La Niña transition from the community as established near the end of the ENSO (May 1998) mainly dominated by *Chenopodium petiolare* (bottom of the plot) to the one established throughout the following La Niña event (August 1998—November 1999) dominated by *Nicotiana paniculata* and *Loasa urens* (top of the biplot). While little change was observed in the species composition in the first two years of our monitoring, there is a clear difference between the composition of the dry and humid months from February 2000 until November 2001 (La Niña event and normal conditions).

**Fig 4:**
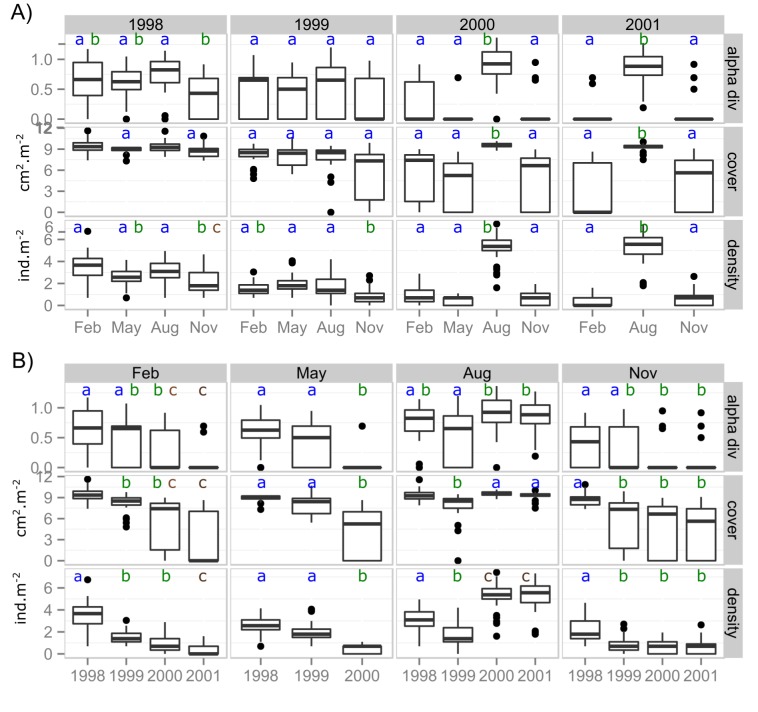
Changes in plant community characteristics. Boxplots of alpha diversity, vegetation cover and density for each sampling campaign between 1998 and 2001 based on 31 plots. A) Boxplots showing temporal changes within years. Months significantly different within each year have different letters (paired t-test with Bonferroni correction, p-value < 0.05). B) Boxplots showing temporal changes for the same month in different years. For each month, the year with values significantly different from other years have different letters (paired t-test with Bonferroni correction, p-value < 0.05). Data has been log-transformed.

The analysis of the plant community characteristics did not show significant differences between the intra-annual values of vegetation cover and alpha diversity during the majority of the 1998 sampling campaigns and all the 1999 sampling campaigns (Fig 4A). Density values showed significant differences between November and August within 1998 and 1999 (p-value <0.05) (Fig 4A). After 1999 values of all the variables for the most humid month (August) were significantly higher than the values for the other months (p-value < 0.01) (Fig 4A).

When we compared the same months throughout the years (Fig 4B) we observed that the values of the plant community characteristics for the dry months (February, May and November) decreased significantly between 1998 and 2001 (p-value < 0.05). The typically most humid month, August, showed a decrease in density values from the year 1998 to 1999 but no significant differences in alpha diversity were found. For the same month, there was a significant increase in the values between 1999 and 2000 for all variables and there were no differences between the values of 2000 and 2001 (Fig 4B).

Finally, gamma diversity values showed oscillations through time with gradual changes (S1 Fig). The amplitude of the oscillations increased after 1999. The oscillations during 1998 and 1999 contrast with the patterns shown by alpha diversity and the other variables where a decreasing trend rather than oscillations were observed for the same period. We did not find any statistically significant differences in the values of plant community characteristics between tourist use zones (S1 Appendix).

### Analysis of changes in vegetation seasonality after the 1997–98 El Niño event

**Fig 5:**
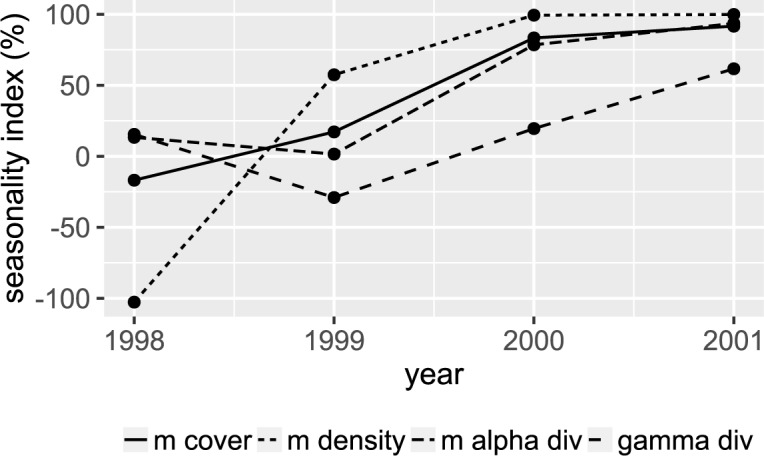
Temporal changes in the seasonality index. The percentage represents the range between the driest and most humid month (February and August respectively) divided by the most humid month (see methods).

Results showed that the seasonality index estimated for density, vegetation cover and alpha diversity (see methods) reached a stable point in the year 2000 where the SI value is similar to that of 2001 (Fig 5 and S5 Table). On the other hand, SI values of gamma diversity do not stabilize in the four years of monitoring and showed an increasing trend. However, by looking at the values of gamma diversity per month we observe that the increase of SI, that represents the ranges between the driest month and the most humid, is dominated by a decrease in the driest month rather than by an increase in the most humid month (S1 Fig).

## Discussion

### Plant community characteristics and climate variables

Except for gamma diversity, all plant community characteristics show a significant correlation with precipitation in our four-year monitoring. The observed positive relationship between biomass (here we used vegetation cover as a proxy) and precipitation is in line with many other studies in drylands [7]. Nevertheless, the observed positive correlation between species richness and precipitation is less clear for different dryland ecosystems. For example, long-term monitoring studies (≥ 5 years) in the west of the Dead Sea [54] and the Chihuahuan desert [55] found the same positive correlation. However, no such correlation was found in a study in southern Peru (Lomas de Mejia) over a one-year monitoring [38]. Instead, our colleagues found a positive correlation between Shannon diversity and water collected from fog [38]. Our study did not implement fog collection measurements, but previous studies found substantial fog contribution in Lomas de Lachay [56]. In other desert regions that have presence of fog such as the Namib desert, the correspondence between species richness and precipitation can vary according to the fog frequency and the amount of rainfall [57]. However, there is little evidence to attribute the difference in the correlation between precipitation and species richness between Lomas de Lachay and Mejia to differences in precipitation patterns, because of a lack of information about latitudinal precipitation patterns along the Peruvian coast [28]. Additionally, other factors can also influence species richness in lomas plant communities such as soil texture and altitude [24,25], which were not evaluated in the present study.

Richness in other dryland ecosystems shows a different relationship with precipitation. For example, a negative relationship was found between precipitation and richness within the dry forest of southern Ecuador [58], but no significant relationship was found in Central American dry forests [59]. In the case of the Ecuadorian dry forest water stress may have led to facilitation processes among species and thus increased richness [58]. However, both studies used vegetation samples across the space from one year. Richness measured after the rainy season in two different years in the northern Peruvian dry forest seems to suggest a positive relationship with precipitation [60].

### Plant community dynamics during the El Niño and La Niña events

#### Seasonality loss and dominant species

The largest impact of the 1997–98 El Niño event on the plant community is the seasonality loss (Figs 3 and 4) during the end of the El Niño and the beginning of the La Niña events (Table 1) evidenced by 1) the constant presence of vegetation cover during 1998 and 1999, and 2) similar species composition from August 1998 and November 1999. We attribute all temporal changes to climate since there was not impact of tourism in the plant community characteristics (S1 Appendix). The atypical presence of vegetation cover, especially herbs, in the normally dry season has been documented before not only in lomas [14,61,62] but also in the northern Peruvian dry forest [18] and in other arid and semi-arid regions [7]. In this study, high atypical precipitation values were recorded only for the first months of 1998, but the vegetation cover persisted continuously until 1999. Plant biomass accumulation is one of the few examples where the pulse-reserve theory [63] can be applied according to our colleagues [1]. In contrast to the pulse-reserve theory, pulse dynamics (no reserve is produced) is more frequent in arid ecosystems for other biological processes such as interactions with other biological communities [1].

A reduced number of species become dominant throughout most of 1998 and 1999 (Fig 3) and most of them have a positive correlation with precipitation. Among these species, *Nicotiana paniculata* clearly dominates the composition together with *Loasa urens* (Figs 2 and 3), an observation that was also made for Lomas de Lachay during the 1982–1983 El Niño [14,62]. The total number of species recorded for Lachay during the El Niño (Feb 1998) was higher than during the beginning of La Niña (Aug 1998–Dec 1999) (S1 Fig). These results are in agreement with those found for Chilean lomas and northern Peruvian dry forest for the same period [16,17].

Surprisingly, although our results show similar species composition towards the end of the El Niño event (Feb 1998) and during the humid months of 2000 (towards the end of La Niña) and 2001 (normal conditions), species richness showed higher values in the latter two sampling campaigns than those recorded during the El Niño event. This could be explained by the presence of dominant species until 1999. In times of high water availability (as is the case during El Niño events), plant-plant competition is hypothesized as the most likely interaction, where dominant species could inhibit other ones by, for example, limiting light availability [64]. The larger individuals of dominant species such as *N*. *paniculata* may have suppressed the seedlings of other species through shading. More studies are required to determine the traits enabling the dominant species to take advantage of the increased water availability.

#### Diversity differences at local and landscape level

Our results also show differences between the temporal patterns of diversity at the plot level (alpha diversity) and at the whole loma level (gamma diversity) especially during the La Niña event (1999). Mean alpha diversity did not show statistically significant differences within 1999 (Fig 4A), but gamma diversity showed oscillations (S1 Fig). This could be due to a change in species richness and evenness, the two components of the Shannon Index used to calculate both diversity values (see methods). For example, although February and May show similar species composition (Fig 3) and similar total richness (Table 1), gamma diversity is higher in February than in May (S1 Fig). This is explained by the higher dominance of *Nicotiana paniculata*, *Loasa urens* and *Nolana humifusa* in May than in February (Fig 2), which affected directly the species evenness, leading to a decrease in gamma diversity. This also implies that in some of the plots the dominant species replaced other ones keeping richness and evenness similar between February and May at the plot level (S2 Table) and, thus, similar values of alpha diversity (Fig 3). The increase in gamma diversity from May to August may result from the opposite pattern previously described: the replacement of the dominant species in some of the plots by other species keeping the same alpha diversity but increasing gamma diversity. Here, population dynamics of dominant species during our monitoring period would be more important in explaining differences between spatial scales [41].

### Re-establishment of the vegetation seasonality

Fogs sustain the Lomas de Lachay ecosystem therefore the seasonal persistence of vegetation cover does not depend on the El Niño event. Therefore, we suggest that in the short term an El Niño event is a disturbance (following definition in [65]) because it changes water resources that leads to the loss of the vegetation seasonality and the reorganization of the plant community. Our results also suggest that the typical annual vegetation seasonality, as per our index estimated for each of the plant community characteristics (Fig 5), and the differences in species composition between the dry and the humid season (Fig 3) were re-established two years after the El Niño event. This suggests that the lomas plant community did not reach a tipping point to change into a perennial shrub community as it has been observed in other arid regions such as parts of the Chihuahuan desert as a consequence of ENSO events before 1995 [66,67]. However, our four-year monitoring is a very small window of time in a system as complex as the lomas ecosystem where extreme ENSO events can occur over decades.

In the long-term, other processes related to the El Niño event may be influencing both, the persistence of the vegetation cover and its species composition [68] in which case the El Niño, rather than a disturbance, could be considered as part of the system. For example, during the El Niño event, lomas ecosystems along the desert coast expand their distribution, allowing populations to connect which promotes genetic exchange [69]. This, together with the normal isolation of the communities and the reduced radiation have been suggested as the most important drivers of the high endemism of the lomas [14,68,70]. Additionally, some desert endemic species depend on the high precipitation levels produced by extreme El Nino events to germinate [71], therefore, one of their main adaptations would be dormancy until these events occur [14]. After the occurrence of an El Niño event, the soil seedbank can increase by a factor of three to five times compared to the normal level [21] leading to the replenishment of the soil seedbank. This way, the renewal of species, renewal of seedbanks and the connection with other populations generated by the El Niño event, may enable this community’s long-term persistence with a diverse and endemic species composition.

### Conservation priorities and future research

The Lomas de Lachay has been a protected area since 1977, therefore the described plant community dynamics during and after ENSO are responses in an environment free of land use change and grazing and where tourism seems not to have affected plant community characteristics variables (S1 Appendix).

Unlike most studies which surveyed study sites in the arid ecosystems of the west coast of South America only during the growing season, we have surveyed our study sites at four points in time throughout the whole annual cycle of vegetation. Thus, our results could be used as baseline for comparisons with lomas that are unprotected and subject to grazing after the El Niño event.

Similar to previous studies [66], our study could provide some guidelines for potential restoration and/or ecosystem adaptation strategies of arid ecosystems after ENSO. For example, we found that the presence of vegetation cover was constant for two years (towards the end of the El Niño and during La Niña), a period that could be used for the management of the wildlife that depends on this vegetation. Also, grazing activities could be restricted in key regions, that are not currently protected, especially during the end of El Niño when high and similar values of alpha diversity occur. The necessity for restoration/management strategies has been made more apparent in the aftermath of the most recent “coastal” El Niño event (February-March 2017), off the northern Peruvian coast, which had precipitation values comparable to that of the 1997–98 ENSO [72]. This presents a real opportunity not only for the above mentioned strategies but also to compare changes in the desert ecosystems to those that occurred in 1997–98 [72].

Lastly, we think that future studies could focus on two important aspects: 1) A framework of interactions between species and plant-resources (competition or cooperation) has been recently suggested to better understand arid ecosystems [1]. This framework could be tested by using our results together with studies that quantified the response of entomofauna [73] and birds [74] to the 1997–98 ENSO. 2) Long-term studies in the Chilean coast suggest that native species would benefit more than invasive ones after a high pulse of precipitation [23], however a study in the Peruvian lomas suggests that invasive species will benefit immediately after a high pulse of rain [34]. The question arises then on how native vs invasive species will respond in the long term, and if there is the danger of invasive species outcompeting native species along the Peruvian Coast.

## Conclusions

This work quantifies the plant community dynamics of a lomas fog oasis, a seasonal community typical of the Peruvian and Chilean desert, during the 1997–98 El Niño event and the subsequent La Niña event. Precipitation during the El Niño event is about three times that of an average month in the humid season. Conversely, the La Niña event, had a mild effect on the lomas, due to less extreme SSTs off the Peruvian coast [42]. Evidence shows that during the 1997–98 El Niño event the high water availability was better utilized by a few species that became dominant. Subsequent changes in species dominance shaped, not only the temporal dynamics of the plant community and its diversity, but also the spatial arrangement of the species. Despite the initial alteration of the species composition, the rapid recovery of the community properties (2 years) suggests a degree of resilience of the plant community that is probably adapted to this recurrent disturbance.

This study, which covered 4 years of continuous survey, provides better understanding of the lomas fragile ecosystem by establishing a comprehensive baseline of the community structure during El Niño, La Niña and normal conditions. Although this provides initial guidelines for restoration and conservation, longer monitoring activities are fundamental to address how recurrent ENSOs have shaped these arid ecosystems and to develop better conservation strategies to protect their unique biodiversity.

## Supporting information

S1 Fig Temporal changes of plant community characteristics and climate variables between 1998 and 2001. Mean values per sampling campaign of vegetation cover (m cover), density (m density), alpha diversity (m alpha div) were obtained by averaging values of all plots per sampling campaign while values of total richness (t richness) and gamma diversity (gamma div) are unique per sampling campaign. Climate variables presented are the monthly mean temperature (Temp.) and the monthly total precipitation (Precip.). Circles in green show referential values (overestimated) for vegetation cover (see methods).
(PDF)

S1 Table Species list. Species recorded in the 15 sampling campaigns of 31 plots of 1m2 in Lomas de Lachay assessed between 1998 and 2001. Nomenclature follows Brako & Zarucchi (1993).
(PDF)

S2 Table Raw abundance and cover data. Species abundance (# individuals) and cover (cm^2^) recorded in the 15 sampling campaigns of 31 plots of 1m2 in Lomas de Lachay assessed between 1998 and 2001.
(XLSX)

S3 Table Spearman correlation between plant community characteristics and climatic variables. We used Bonferroni correction for multiple comparisons. Correlation coefficients (Rho), original p-values (p_value) and p values after the correction are shown (p_value_bonf).
(PDF)

S4 Table Spearman correlation between species density and climatic variables. We used Bonferroni correction for multiple comparisons. Correlation coefficients (Rho), original p-values (p_value) and p values after the correction are shown (p_value_bonf).
(PDF)

S5 Table Seasonality index (SI) for each plant community characteristic. The index is calculated by using the values recorded for the typical driest month (February) and the typical most humid month (August). See methods for a full description.
(PDF)

S1 Appendix Kruskal–Wallis test comparing plant community characteristics between tourist use zones. Analysis for alpha diversity, vegetation cover and density.
(PDF)
