# Supplementary figures and images for: Plant community dynamics of lomas fog oasis of Central Peru after the extreme precipitation caused by the 1997-98 El Niño event
Source: PLoS One. 2018 Jan 2;13(1):e0190572. doi: 10.1371/journal.pone.0190572 (PMC5749840; doi:10.1371/journal.pone.0190572)

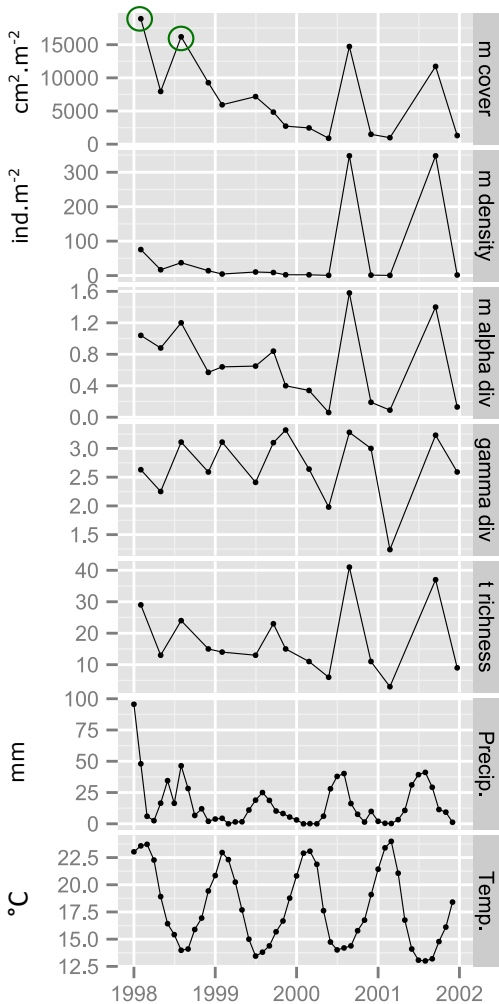

Supplement: S1 Fig — Mean values per sampling campaign of vegetation cover (m cover), density (m density), alpha diversity (m alpha div) were obtained by averaging values of all plots per sampling campaign while values of total richness (t richness) and gamma diversity (gamma div) are unique per sampling campaign. Climate variables presented are the monthly mean temperature (Temp.) and the monthly total precipitation (Precip.). Circles in green show referential values (overestimated) for vegetation cover (see methods). (PDF) [file pone.0190572.s001.pdf]
