# Supplementary material for: Plant community dynamics of lomas fog oasis of Central Peru after the extreme precipitation caused by the 1997-98 El Niño event
Source: PLoS One. 2018 Jan 2;13(1):e0190572. doi: 10.1371/journal.pone.0190572 (PMC5749840; doi:10.1371/journal.pone.0190572)
Supplement: S1 Table — Species recorded in the 15 sampling campaigns of 31 plots of 1m2 in Lomas de Lachay assessed between 1998 and 2001. Nomenclature follows Brako & Zarucchi (1993). (PDF) [file pone.0190572.s002.pdf]

S1 Table. Species list. Species recorded in the 15 sampling campaigns of 31 plots of 1m^2 in Lomas de Lachay assessed between 1998 and 2001. Nomenclature follows Brako & Zarucchi (1993).

Family	Species
Acanthaceae	*Dicliptera sp*
Acanthaceae	*Dyschoriste repens* (Nees) Kuntze
Amaranthaceae	*Alternanthera halimifolia* (Lam.) Standl. ex Pittier
Amaryllidaceae	*Furcraea andina* Trel.
Amaryllidaceae	*Stenomesson coccineum* Herb.
Apiaceae	*Bowlesia palmata* Ruiz & Pav.
Asteraceae	*Acmella oleracea* (L.) R.K. Jansen
Asteraceae	*Ageratina sternbergiana* (DC.) R.M. King & H. Rob.
Asteraceae	*Cotula australis* (Sieber ex Spreng.) Hook. f.
Asteraceae	*Erigeron leptorhizon* DC.
Asteraceae	*Galinsoga sp*
Asteraceae	*Gnaphalium dombeyanum* DC.
Asteraceae	*Ophryosporus peruvianus* (J.F. Gmel.) R.M. King & H. Rob.
Asteraceae	*Philoglossa peruviana* DC.
Asteraceae	*Senecio lomincola* Cabrera
Asteraceae	*Siegesbeckia flosculosa* L'Hér.
Asteraceae	*Sonchus oleraceus* L.
Asteraceae	*Trixis cacalioides* (Kunth) D. Don
Asteraceae	*Vasquezia oppositifolia* (Lag.) S.F. Blake
Bignoniaceae	*Tourrettia lappacea* (L'Hér.) Willd.
Boraginaceae	*Heliotropium angiospermum* Murray
Boraginaceae	*Heliotropium arborescens* L.
Cactaceae	Sp1
Caryophyllaceae	*Drymaria divaricata* Kunth
Chenopodiaceae	*Chenopodium petiolare* Kunth
Convolvulaceae	*Evolvulus villosus* Ruiz & Pav.
Crassulaceae	*Crassula connata* (Ruiz & Pav.) A. Berger
Cucurbitaceae	*Sicyos baderoa* Hook. & Arn.
Euphorbiaceae	*Croton ruizianus* Müll. Arg.
Hydrophyllaceae	*Nama dichotoma* (Ruiz & Pav.) Choisy
Lamiaceae	*Hyptis sidifolia* (L'Hér.) Briq.
Lamiaceae	*Salvia rhombifolia* Ruiz & Pav.
Liliaceae	*Fortunatia biflora* (Ruiz & Pav.) J.F. Macbr.
Loasaceae	*Loasa urens* Jacq.
Malvaceae	*Urocarpidium peruvianum* (L.) Krapov.
Onagraceae	*Oenothera rosea* L'Hér. ex Aiton
Oxalidaceae	*Oxalis bulbigera* R. Knuth
Poaceae	*Andropogon sp*
Poaceae	*Avena barbata* Pott ex Link
Poaceae	*Cenchrus ciliaris* L.
Poaceae	*Cynodon sp*
Poaceae	*Eragrostis mexicana* (Hornem.) Link

Poaceae	*Lophochloa cristata* (L.) Hyl.
Poaceae	*Paspalum flavum* J. Presl
Poaceae	Poaceae Barnhart
Portulacaceae	*Calandrinia alba* (Ruiz & Pav.) DC.
Portulacaceae	*Calandrinia sp*
Portulacaceae	*Cistanthe sp*
Scrophulariaceae	*Calceolaria pinnata* L.
Solanaceae	*Lycopersicon peruvianum* (L.) Mill.
Solanaceae	*Lycopersicon pimpinellifolium* (L.) Mill.
Solanaceae	*Nicotiana paniculata* L.
Solanaceae	*Nolana humifusa* (Gouan) I.M. Johnst.
Solanaceae	*Solanum montanum* L.
Solanaceae	*Solanum phyllanthum* Cav.
Sterculiaceae	*Waltheria ovata* Cav.
Urticaceae	*Parietaria debilis* G. Forst.
Urticaceae	*Urtica urens* L.
Valerianaceae	*Astrephia chaerophylloides* (Sm.) DC.
Verbenaceae	*Lippia nodiflora* (L.) Michx.

References

Brako, L., & Zarucchi, J.L. 1993. Catalogue of the flowering plants and gymnosperms of Peru. Missouri Botanical Garden.
