# Supplementary material for: Plant community dynamics of lomas fog oasis of Central Peru after the extreme precipitation caused by the 1997-98 El Niño event
Source: PLoS One. 2018 Jan 2;13(1):e0190572. doi: 10.1371/journal.pone.0190572 (PMC5749840; doi:10.1371/journal.pone.0190572)
Supplement: S3 Table — We used Bonferroni correction for multiple comparisons. Correlation coefficients (Rho), original p-values (p_value) and p values after the correction are shown (p_value_bonf). (PDF) [file pone.0190572.s004.pdf]

S3 Table. Spearman correlation between plant community characteristics and climatic variables. We used Bonferroni correction for multiple comparisons. Correlation coefficients (Rho), original p-values (p_value) and p values after the correction are shown (p_value_bonf).

Rho	p_value	qf	p_value_bonf	variable
0.818	0.0003	Mean density	**0.0018**	Total monthly precipitation
0.804	0.0005	Mean vegetation cover	**0.0029**	Total monthly precipitation
0.839	0.0001	Mean alpha diversity	**0.0006**	Total monthly precipitation
0.386	0.1548	Gamma diversity	0.9290	Total monthly precipitation
0.806	0.0002	Total richness	**0.0017**	Total monthly precipitation
-0.368	0.1779	Mean density	1	Mean monthly temperature
-0.229	0.4114	Mean vegetation cover	1	Mean monthly temperature
-0.432	0.109	Mean alpha diversity	0.6565	Mean monthly temperature
-0.447	0.0946	Gamma diversity	0.5678	Mean monthly temperature
-0.408	0.1309	Total richness	0.7853	Mean monthly temperature
