# Supplementary material for: Plant community dynamics of lomas fog oasis of Central Peru after the extreme precipitation caused by the 1997-98 El Niño event
Source: PLoS One. 2018 Jan 2;13(1):e0190572. doi: 10.1371/journal.pone.0190572 (PMC5749840; doi:10.1371/journal.pone.0190572)
Supplement: S4 Table — We used Bonferroni correction for multiple comparisons. Correlation coefficients (Rho), original p-values (p_value) and p values after the correction are shown (p_value_bonf). (PDF) [file pone.0190572.s005.pdf]

S4 Table. Spearman correlation between species density and climatic variables. We used Bonferroni correction for multiple comparisons. Correlation coefficients (Rho), original p-values (p_value) and p values after the correction are shown (p_value_bonf).

Rho	p_value	qf	p_value_bonf	variable
0.597	0.019	*Acmella oleracea*	0.355	Total monthly precipitation
0.096	0.734	*Alternanthera halimifolia*	1	Total monthly precipitation
0.586	0.022	*Chenopodium petiolare*	0.410	Total monthly precipitation
-0.249	0.370	*Croton ruizianus*	1	Total monthly precipitation
0.498	0.059	*Hyptis sidifolia*	1	Total monthly precipitation
0.701	0.004	*Loasa urens*	0.068	Total monthly precipitation
0.130	0.644	*Lycopersicon peruvianum*	1	Total monthly precipitation
0.779	0.001	*Nicotiana paniculata*	**0.019**	Total monthly precipitation
0.726	0.002	*Nolana humifusa*	**0.041**	Total monthly precipitation
0.343	0.211	*Ophryosporus peruvianus*	1	Total monthly precipitation
0.582	0.023	*Parietaria debilis*	0.433	Total monthly precipitation
0.827	0.000	*Poaceae*	**0.003**	Total monthly precipitation
0.854	0.000	*Senecio lomincola*	**0.001**	Total monthly precipitation
0.763	0.001	*Solanum phyllantum*	**0.018**	Total monthly precipitation
0.400	0.139	*Stenomesson coccineum*	1	Total monthly precipitation
0.729	0.002	*Sycios baderoa*	**0.039**	Total monthly precipitation
-0.450	0.092	*Trixis cacalioides*	1	Total monthly precipitation
0.732	0.002	*Urocarpidium peruvianum*	**0.036**	Total monthly precipitation
0.782	0.001	*Vasquezia oppositifolia*	**0.011**	Total monthly precipitation
-0.276	0.319	*Acmella oleracea*	1	Mean monthly temperature
0.011	0.968	*Alternanthera halimifolia*	1	Mean monthly temperature
0.058	0.838	*Chenopodium petiolare*	1	Mean monthly temperature
0.004	0.990	*Croton ruizianus*	1	Mean monthly temperature
0.058	0.836	*Hyptis sidifolia*	1	Mean monthly temperature
-0.598	0.019	*Loasa urens*	0.352	Mean monthly temperature
0.487	0.066	*Lycopersicon peruvianum*	1	Mean monthly temperature
-0.421	0.119	*Nicotiana paniculata*	1	Mean monthly temperature
-0.552	0.033	*Nolana humifusa*	0.628	Mean monthly temperature
0.072	0.798	*Ophryosporus peruvianus*	1	Mean monthly temperature
-0.655	0.008	*Parietaria debilis*	0.152	Mean monthly temperature
-0.578	0.024	*Poaceae*	0.458	Mean monthly temperature
-0.569	0.027	*Senecio lomincola*	0.507	Mean monthly temperature
-0.325	0.237	*Solanum phyllantum*	1	Mean monthly temperature
-0.373	0.171	*Stenomesson coccineum*	1	Mean monthly temperature
-0.438	0.103	*Sycios baderoa*	1	Mean monthly temperature
0.350	0.201	*Trixis cacalioides*	1	Mean monthly temperature
-0.482	0.069	*Urocarpidium peruvianum*	1	Mean monthly temperature
-0.400	0.140	*Vasquezia oppositifolia*	1	Mean monthly temperature
