# Supplementary material for: Plant community dynamics of lomas fog oasis of Central Peru after the extreme precipitation caused by the 1997-98 El Niño event
Source: PLoS One. 2018 Jan 2;13(1):e0190572. doi: 10.1371/journal.pone.0190572 (PMC5749840; doi:10.1371/journal.pone.0190572)
Supplement: S5 Table — The index is calculated by using the values recorded for the typical driest month (February) and the typical most humid month (August). See methods for a full description. (PDF) [file pone.0190572.s006.pdf]

S5 Table. Seasonality index (SI) for each plant community characteristic. The index is calculated by using the values recorded for the typical driest month (February) and the typical most humid month (August). See methods for a full description.

Variable	Year	February	August	SI
mean vegetation cover	1998	18911.8	16184.7	-16.8
	1999	5943.9	7175.9	17.2
	2000	2451.6	14720.8	83.3
	2001	983.5	11728.1	91.6
mean density	1998	75.5	37.3	-102.7
	1999	4.4	10.4	57.4
	2000	2.2	347.7	99.4
	2001	0.5	347.6	99.9
mean alpha diversity	1998	1.0	1.2	13.3
	1999	0.6	0.7	1.5
	2000	0.3	1.6	78.5
	2001	0.1	1.4	93.6
gamma diversity	1998	2.6	3.1	15.4
	1999	3.1	2.4	-29.0
	2000	2.6	3.3	19.5
	2001	1.2	3.2	61.6
