# Supplementary material for: Plant community dynamics of lomas fog oasis of Central Peru after the extreme precipitation caused by the 1997-98 El Niño event
Source: PLoS One. 2018 Jan 2;13(1):e0190572. doi: 10.1371/journal.pone.0190572 (PMC5749840; doi:10.1371/journal.pone.0190572)
Supplement: S1 Appendix — Analysis for alpha diversity, vegetation cover and density. (PDF) [file pone.0190572.s007.pdf]

S1 Appendix. Kruskal–Wallis test comparing plant community characteristics between tourism use zones. Analysis for alpha diversity, vegetation cover and density.

We used the non-parametric Kruskal-Wallis test to check whether values of alpha diversity, cover and vegetation cover per plot were different between tourist use zones. We used the function kruskal.test in R.

Mean values of the plant community characteristics per tourist use zone for all dataset

First, we plot the mean values of each plant community characteristic per tourist use zone, together with their confidence intervals, to have a visualization of the values. Then, we used a dataset of 465 records (15 fieldwork campaigns x 31 plots) for each of the plant community characteristics to perform the Kruskal-Wallis test. As this is an analysis that requires absolute values we excluded the fieldwork sampling campaigns of January and August 1998 for vegetation cover due to the overestimation of this measurement for those dates (see methods). Results showed no statistically significant differences in alpha diversity, density and vegetation cover between tourist use zones.

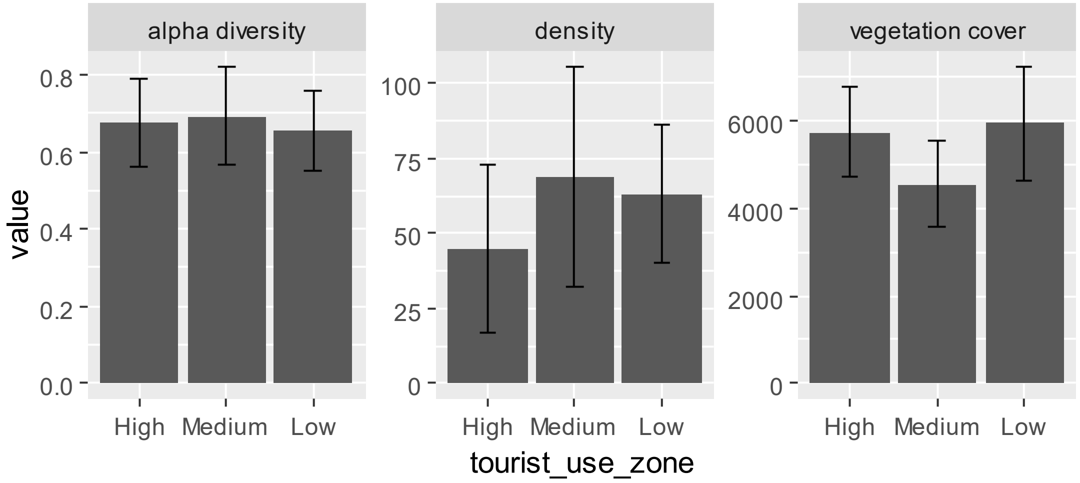

Data	Kruskal-Wallis chi-squared	df	p-value
alpha_div by tourist_use_zone	0.1724	2	0.9174
density by tourist_use_zone	0.55321	2	0.7584
cover by tourist_use_zone	1.1592	2	0.5601

Mean values of the plant community characteristics per tourist use zone for each sampling campaign

Second, we plotted mean values and confidence intervals for alpha diversity, density and vegetation cover per tourist use zone per sampling campaign. Then, we performed the same Kruskal-Wallis test per sampling campaign (31 records for each test). Because this were tests within fieldwork campaigns we included the values of January and August 1998 for the analysis of vegetation cover. We used the Bonferroni correction to adjust p-values for each of the plant community characteristics due to the multiple tests that were performed. We did not find any statistically significant difference in alpha diversity, vegetation cover and density between tourist use zones for each sampling campaign.

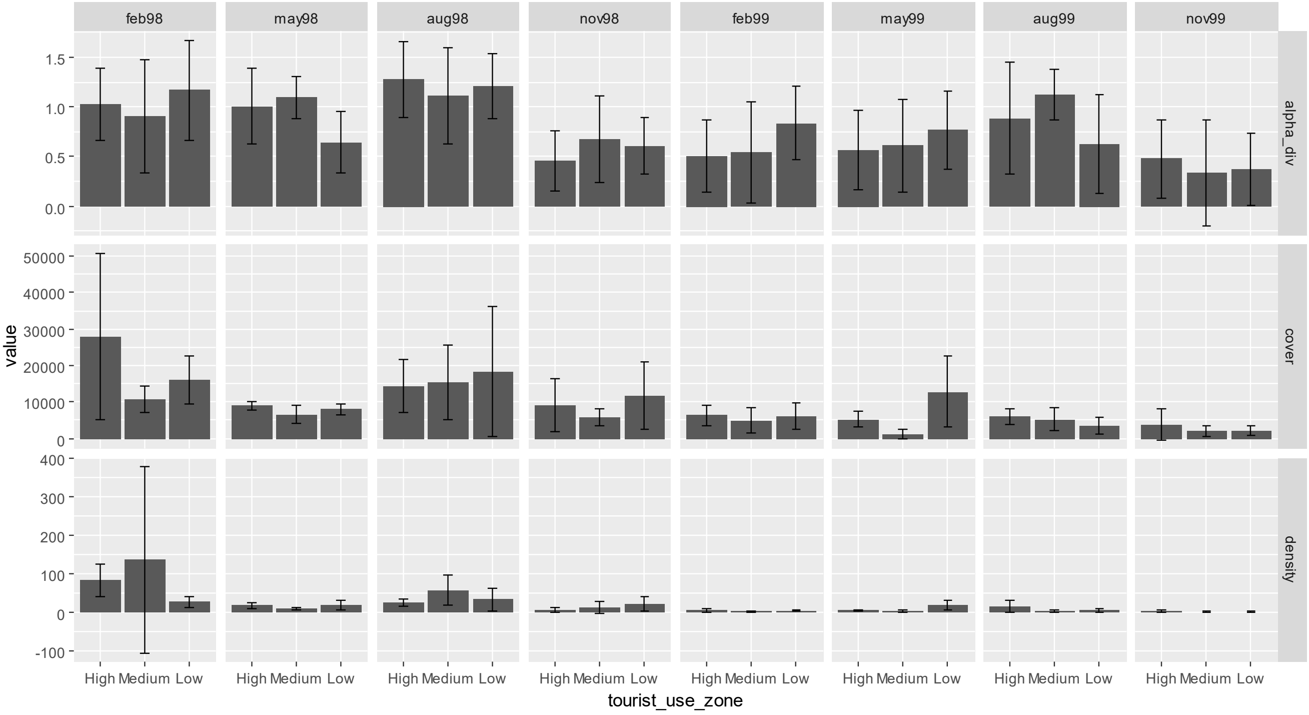
feb98
may98
aug98
nov98
feb99
may99
aug99
nov99
alpha_div
cover
density
value
tourist_use_zone
High Medium Low

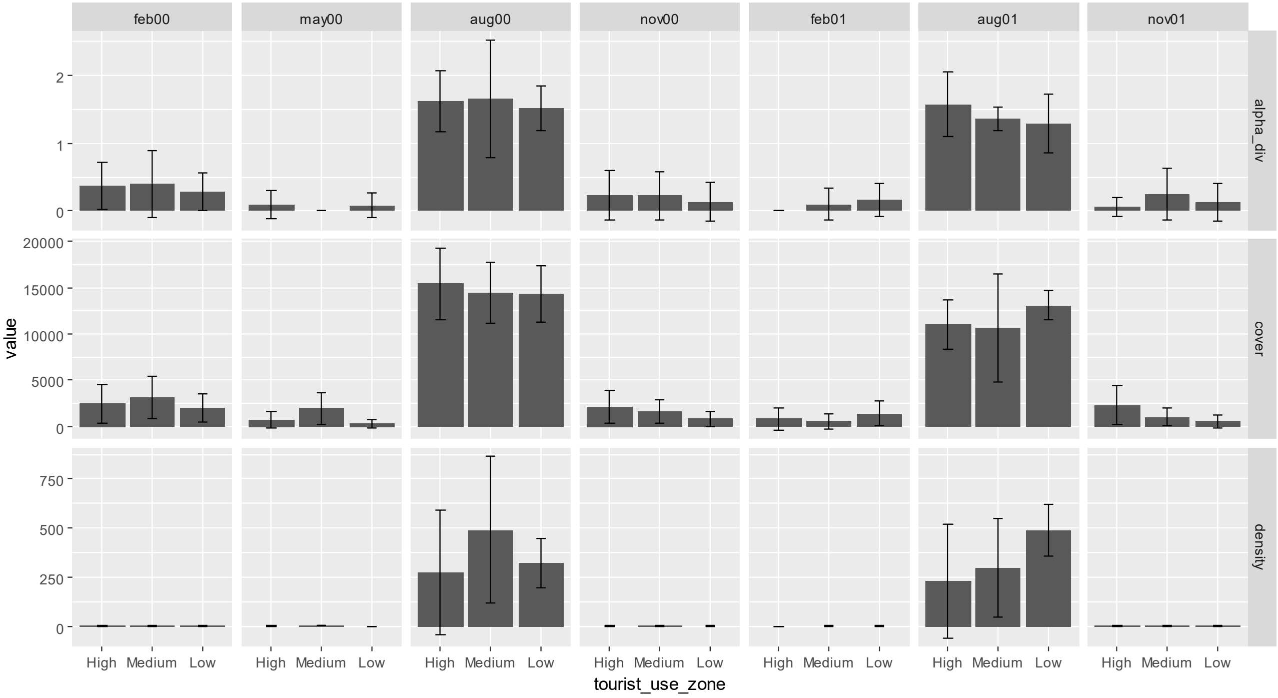
feb00
may00
aug00
nov00
feb01
aug01
nov01
alpha_div
cover
density
value
tourist_use_zone
High Medium Low

variable	fieldwork campaign	chi_squared	df	p_value	p_adjust
Alpha diversity	Feb-98	0.874	2	0.646	1
	May-98	5.409	2	0.067	1
	Aug-98	1.154	2	0.562	1
	Nov-98	0.869	2	0.648	1
	Feb-99	2.304	2	0.316	1
	May-99	0.874	2	0.646	1
	Aug-99	1.994	2	0.369	1
	Nov-99	0.384	2	0.825	1
	Feb-00	0.325	2	0.850	1
	May-00	0.725	2	0.696	1
	Aug-00	0.340	2	0.844	1
	Nov-00	0.719	2	0.698	1
	Feb-01	1.925	2	0.382	1
	Aug-01	1.153	2	0.562	1
	Nov-01	1.356	2	0.508	1
density	Feb-98	4.930	2	0.085	1
	May-98	1.496	2	0.473	1
	Aug-98	4.079	2	0.130	1
	Nov-98	3.621	2	0.164	1
	Feb-99	2.155	2	0.340	1
	May-99	5.372	2	0.068	1
	Aug-99	1.056	2	0.590	1
	Nov-99	0.136	2	0.934	1
	Feb-00	0.197	2	0.906	1
	May-00	5.868	2	0.053	0.798
	Aug-00	5.049	2	0.080	1
	Nov-00	3.908	2	0.142	1
	Feb-01	0.074	2	0.964	1
	Aug-01	10.950	2	0.004	0.063
	Nov-01	2.071	2	0.355	1
cover	Feb-98	0.840	2	0.657	1
	May-98	4.774	2	0.092	1
	Aug-98	0.514	2	0.773	1
	Nov-98	0.480	2	0.787	1
	Feb-99	0.805	2	0.669	1
	May-99	9.932	2	0.007	0.105
	Aug-99	3.390	2	0.184	1
	Nov-99	0.022	2	0.989	1
	Feb-00	1.216	2	0.545	1
	May-00	9.204	2	0.010	0.150
	Aug-00	0.211	2	0.900	1
	Nov-00	2.619	2	0.270	1
	Feb-01	0.095	2	0.954	1
	Aug-01	3.258	2	0.196	1
	Nov-01	3.184	2	0.203	1
